# Effects of a Covert Infection with Phthorimaea operculella granulovirus in Insect Populations of *Phthorimaea operculella*

**DOI:** 10.3390/v11040337

**Published:** 2019-04-09

**Authors:** Andreas Larem, Saoussen Ben Tiba, Eva Fritsch, Karin Undorf-Spahn, Jörg T. Wennmann, Johannes A. Jehle

**Affiliations:** Institute for Biological Control, Federal Research Centre for Cultivated Plants, Julius Kühn Institute, Heinrichstraße 243, 64287 Darmstadt, Germany; Andreas.Larem@julius-kuehn.de (A.L.); sawssen.bentiba@gmail.com (S.B.T.); eva.fritsch@julius-kuehn.de (E.F.); Karin.undorf-spahn@julius-kuehn.de (K.U.-S.); joerg.wennmann@julius-kuehn.de (J.T.W.)

**Keywords:** PhopGV, *Baculoviridae*, *Betabaculovirus*, potato tuber moth, genome sequencing, sublethal infection, co-infection

## Abstract

Virus infections of insects can easily stay undetected, neither showing typical signs of a disease, nor being lethal. Such a stable and most of the time covert infection with Phthorimaea operculella granulovirus (PhopGV) was detected in a *Phthorimaea operculella* laboratory colony, which originated from Italy (Phop-IT). This covert virus (named PhopGV-R) was isolated, purified and characterized at the genetic level by full genome sequencing. Furthermore, the insect colony Phop-IT was used to study the crowding effect, double infection with other PhopGV isolates (CR3 and GR1), and co-infection exclusion. An infection with a second homologous virus (PhopGV-CR3) activated the covert virus, while a co-infection with another virus isolate (PhopGV-GR1) led to its suppression. This study shows that stable virus infections can be common for insect populations and have an impact on population dynamics because they can suppress or enable co-infection with another virus isolate of the same species.

## 1. Introduction

Virus transmission can follow two different mechanisms. An overt virus outbreak can kill a large number of individuals of a population and may function as an infective source for other host insects, either from the same or another generation (horizontal transmission); or a virus infection does not result in the death of the infected individual and can be transmitted from the infected parental generation to their offspring (vertical transmission) [1]. Horizontal virus transmission conveyed by per os infection initiating an acute virus infection cycle is well characterized for many insect viruses, including nudiviruses and baculoviruses [2,3]. However, the understanding of the mechanisms involved in vertical virus transmission, as observed in some cases for rhabdoviruses (e.g., sigma virus) and picornaviruses (e.g., *Dicistroviridae*) [4,5], is still in its infancy [6]. In contrast to acute infection after horizontal transmission, virus infections exploiting vertical transmission are often covert and chronic. Such covert infections can appear either as persistent infections resulting in a continuous low level of virus replication after a primary infection, or as a latent infection when the primary infection is followed by a low level reactivation and recurrent infection [7]. The verification of covert infections is complicated, because the time point of analysis is crucial and can be misleading.

Covert infections have been an intriguing field of research for viruses of the family *Baculoviridae*. Baculoviruses are insect-specific dsDNA viruses associated with mainly larval stages of insects from the orders Lepidoptera (genera *Alphabaculovirus* and *Betabaculovirus*), Hymenoptera (genus *Gammabaculovirus*) and Diptera (genus *Deltabaculovirus*) [8]. Whereas overt infections result in heavily diseased larvae which succumb to baculovirus infection, covert infections can be survived and become stably established in an insect population [9], characterized by the absence of visible signs of infection [10]. Covert baculovirus infections can be found in many different insect populations in the laboratory and in the field [6,11,12,13,14,15]. One method to obtain new baculoviruses is to collect insects from field populations and rear them in the laboratory until a virus outbreak occurs. This method has been successfully applied to isolate betabaculoviruses from various insects and environments, such as South-African strains of Phthorimaea operculella granulovirus (PhopGV-SA), Plutella xylostella granulovirus (PlxyGV-SA) and Cryptophlebia leucotreta granulovirus (CrleGV-SA) [16,17,18]. The latter was obtained after overcrowding larvae of a reared *Thaumatotibia leucotreta* colony. There are also many examples for alphabaculovirus outbreaks in insect colonies which lead to successful isolations of new virus samples, e.g., Mamestra brassicae multiple nucleopolyhedrovirus (MbMNPV), Trichoplusia ni single nucleopolyhedrovirus (TnSNPV) and Spodoptera exigua multiple nucleopolyhedrovirus (SeMNPV) [11,13,19,20].

Baculoviruses can regulate insect populations [21,22,23], and it is therefore of eminent interest to consider the natural occurring viruses present in dynamic insect populations. Overt infections are rare events in insect field populations and often correlate with mass outbreaks of the host insects [24,25]. Horizontal virus transmission is an effective viral strategy for host species with high population densities, but for low host densities, such a transmission strategy would bear the risk for a given virus of becoming extinct [26]. Vertical transmission is a long-term strategy for persistence in host populations [13] and is linked to low virulence [10]. However, these low-virulence covert infections can be triggered by stressors like temperature, humidity, nutrition, overcrowding or a secondary infection with a homologous or heterologous virus [13,14,20,27,28,29]. Evolutionary success of baculoviruses is closely related to a mixed-mode strategy involving both horizontal and vertical transmission that is very common across a broad range of viruses, parasites, symbionts, and microbiota [30].

Phthorimaea operculella granulovirus (PhopGV) (genus *Betabaculovirus,* family *Baculoviridae*) is a dsDNA virus with a circular genome of about 119 kb, encoding 130 open reading frames (ORF), [16,31]. It can infect several members of the lepidopteran family Gelechiidae like *Phthorimaea operculella*, *Tuta absoluta*, *Tecia solanivora* and others [32,33,34]. Recently, a comprehensive comparison of 14 genome sequences of different PhopGV has been published, resulting in the identification of four phylogenetic lineages which also corresponded to the distribution and prevalence of single nucleotide polymorphisms and insertion and deletion (indels) mutations [35]. The virus-encoded gene *ecdysteroid UDP-glucosyl transferase* (*egt*, ORF129) is of particular interest, because its gene expression leads to the inactivation of ecdysteroid, which acts as a molting hormone in host development [36,37]. In the PhopGV genome, the *egt* gene can occur in five different size variants, with ORF lengths between 861 and 1353 bp [17]. This variability of the *egt* gene has also been used to group and differentiate PhopGV isolates according to these five *egt* types (I-V) [17,32]. Another highly variable gene is *superoxide dismutase* (*sod*, ORF54) [35].

In this study, we characterized a new PhopGV isolated from a persistent covert infected laboratory rearing of *P. operculella* (Phop-IT). This infection was covert in most cases, which means that no virus-killed individuals were detected, but a potential for an active overt virus outbreak was present. In addition, the infection was persistent over many generations. Transmission of virus from parents to the next following generation was most likely carried out through eggs, where a transovarial or transovum pathway is possible. Transovarial means a transmission from maternal parent to progeny embryos within the eggs, whereas transovum involves contamination of the exterior egg surface with viral particles that infect neonate larvae as they ingest the chorion during hatching (eclosion) [6,10,22]. We further studied the effects of this virus on the insect population in terms of development and susceptibility for secondary virus infections. We showed that a mixed mode of interaction is possible, and the covert virus can either be activated by a secondary virus and becomes overt as a mix of both viruses, or can be suppressed by other virus strains within the same virus species. Activation and/or suppression of covert viruses, as found with PhopGV, are crucial for the understanding of virus population dynamics and viral ecology. In addition, they argue for a careful quality management when baculoviruses are produced as biological control agents under in vivo conditions.

## 2. Materials and Methods

### 2.1. Insects

A rearing of the potato tuber moth *Phthorimaea operculella* was established at JKI in 2014. The colony was obtained from COOP. TERREMERSE, Bagnacavallo (Italy) and was based on insects collected in the Emilia Romagna, Ravenna. This strain was named Phop-IT. Organic grown potatoes, *Solanum tuberosum,* were used as diet. The larvae were kept on potato slices at 26 °C and under 16/8 h light/dark conditions until pupation. Potato slices were placed on sand to allow pupation outside of the potato and to ease the collection of the pupa with a mesh. After hatching, the adults were transferred into cylinders (ᴓ 24.5 cm, height = 18 cm), covered with a dark plastic bag, and placed on a tray. A piece of cotton wool, watered with 10% sucrose solution, was provided to feed adults. The top end of the cylinder was closed with fine gauze, which was permeable for egg laying on a filter paper placed on top of the gauze outside of the cylinder. This technique allowed an exchange of the egg paper with a fresh one without opening the cylinder. After the collection of the egg paper, the eggs were incubated at either 20 °C or 26 °C, which allowed manipulating the hatching day of the neonate larvae. Sterile material was used for the rearing in order to avoid microbial contamination. To eliminate the risk of contamination across generations, single use material was applied.

### 2.2. Viruses

Different isolates of the Phthorimaea operculella granulovirus were used in this study: (i) PhopGV-R (covert virus of the Phop-IT rearing), (ii) PhopGV-CR3.1 (origin Costa Rica) [38], and (iii) PhopGV-GR1.1 (field collection; Greece). PhopGV-CR3.1 and PhopGV-GR1.1 were the first larval passages of OB samples of isolates PhopGV-CR3 (Public University of Navarra, Agrobiotechnology Institute, Pamplona, Spain), and PhopGV-GR1 (Hellafarm SA, Attica, Greece), respectively. Larval passages were conducted with the laboratory colony Phop-IT. The propagated PhopGV isolates PhopGV-CR3 and PhopGV-GR1 received the suffix “.1” to indicate their passage through the host Phop-IT during virus propagation. Propagation of original virus sample was necessary to obtain a sufficient viral occlusion body (OB) stock for the following experiments.

### 2.3. Co-Propagation of PhopGV-CR3 and PhopGV-GR1

The surface of each potato disc (4.3 cm Ø, 0.5 cm thickness) was inoculated with 200 µL OB suspension (1 × 10^4^ OB/mL) of either PhopGV-CR3 or PhopGV-GR1. Twenty neonate larvae of *P. operculella* were transferred onto one potato disc using a fine paint brush. The potato discs were kept at 26 °C, 60% RH, and under a 16/8 h light/dark regime. After six days, the inoculated larvae showed typical baculovirus infection symptoms, such as loss of mobility, decreased feeding rate and change in color from green to bright white, followed by sluggishness and flaccidity [39,40,41,42]. From this point, the potato discs were examined daily, and dead larvae were collected. The experiment was repeated until a total of 80 virus-killed larvae of each virus treatment was obtained.

### 2.4. Virus Purification

For the homogenization of the pooled larval material, an electric homogenizer (IKA T25 digital Ultra Turrax) was used. The purification of the viral occlusion bodies (OB) followed a modified protocol of Smith and Crook [43]. The obtained homogenate was treated with sodium dodecyl sulphate (SDS) (10% *w*/*v*) to a final concentration of 0.5% and kept on ice for 30 min. After 3 min incubation in an ultrasonic water bath, the sample was filtered through a sandwich filter made of cotton and cotton wool. To minimize the loss of viral OBs, the filter was washed with additional volumes of 0.5% SDS or dH_2_O. After several washing steps, including centrifugation at 22,000× *g* for 15 min and re-suspension of the pellet with dH_2_O, larval debris was removed from the OB sample. The obtained OB suspension was loaded onto a glycerol step gradient: 50%, 55%, 60%, 70%, and 80% (*v*/*v*) top to bottom in 15 mL tubes. Prepared step gradients were loaded into an A-4-62 swing-out rotor and centrifuged at 3200 g for 45 min in an Eppendorf 5810R centrifuge. OBs were visible as a light grey band between 55% and 60% glycerol concentration. This band was collected and washed with dH_2_O by additional centrifugation steps to remove the glycerol from the sample.

### 2.5. DNA Isolation from Occlusion Body

Separately for each isolate, PhopGV-R, PhopGV-CR3.1 and PhopGV-GR1.1, 150 µL of purified OB suspension (1.6 × 10^12^ OB/mL) derived from infected Phop-IT larvae were used for DNA isolation, according to the protocol of Arends and Jehle [44].

### 2.6. Whole DNA Isolation from P. operculella Eggs, Larvae, Pupae and Adults

DNA isolation from variously sized batches of eggs (*n* = 5, 10, 15, 20 and 30), individual larvae (*n* = 150), individual pupae (*n* = 50), and individual adults (*n* = 50) was performed by following a slightly modified protocol of Ron’s tissue DNA Mini Kit (Bioron GmbH, Ludwigshafen, Germany). First, samples were ground thoroughly in the kit’s lysis buffer using a mortar and pestle. Then, the obtained homogenates were centrifuged in 1.5 mL tubes for 2 min at maximum speed (20,800× *g*) in an Eppendorf 5417R centrifuge to remove any larval debris. Supernatants were used for total DNA purification following the manufacturer’s instructions.

### 2.7. Complete egt Gene Amplification

The oligonucleotide primer pair egtF (5’-GAG TCG AGCCAA TTT TGT TTG CG-3’) and egtR (5’-GCA ACG ATG ATC TCATAT ATG AGC-3’) [17], flanking 204 bp up- and downstream ORF 129, were used for PCR amplification of the *ecdysteroid UDP–glucosyltransferase (egt)* gene region. The reactions comprised 10 µL of 5× Green Buffer (including MgCl_2_ 7.5 mM), 1 µL dNTPs (10 mM each), 1 µL of forward and reverse primer (10 µM), respectively, 50 ng DNA template and 0.25 µL Go Taq Polymerase (5 U) (Promega GmbH, Mannheim, Germany). Reactions were filled up to a final volume of 50 µL using ddH_2_O. PCR reactions were initiated by a denaturation step at 94 °C for 4 min, followed by 33 cycles of 94 °C for 1 min, 52 °C for 1 min, 72 °C for 1 min and a final extension step at 72 °C for 5 min. Amplified products were visualized by agarose gel (1%) electrophoresis at 90 V for 45 min in 1× TAE buffer (40 mM Tris-acetate, 20 mM acetic acid, 1mM EDTA) stained with Midori Green Advance (Nippon Genetics Europe GmbH, Dueren, Germany). Visualization and caption of the gel images were done with ChemoCam Imager ECL UV trans-illuminator and software (INTAS Science Imaging Instruments GmbH, Göttingen, Germany).

### 2.8. DNA Restriction Endonuclease Digests

The PCR amplification products were purified using the Clean & Concentrator Kit-25 (Zymo Research Europe GmbH, Freiburg, Germany). DNA concentration and quality were determined by measuring 1 µL of the DNA samples in a Nanodrop 2000c Spectrometer (Thermo Scientific, Wilmington, Delaware USA). DNA restriction endonuclease (REN) digests comprised 17.5 µL DNA in dH_2_O (800 ng), 2 µL reaction buffer, 0.2 µL of 10 µg/µL BSA and 0.5 µL AluI (Promega GmbH, Mannheim, Germany). The reaction was incubated for 5.5 h at 37 °C and loaded directly on a 1% agarose (TAE) gel.

### 2.9. Mixed Infection of Phop-IT Neonate Larvae

For setting up infection experiments, 200 µL of a PhopGV OB suspension (1.3 × 10^4^ OB/mL) were applied to inoculate the surface of a potato disc (ᴓ 43 mm and 5 mm thickness). Either PhopGV-R or an equal mixture of 100 µL PhopGV-R and 100 µL PhopGV-GR1.1 was applied as inoculum. By using a knife, several parallel cuts were applied to the surface of the potato slices in order to facilitate the finding of the test larvae at 7 days post inoculation (dpi) and 14 dpi. Twenty neonate larvae were placed on each potato slice using a fine brush. Each of the potato slices was kept in a Petri dish and incubated at 26 °C with a 16/8 h light/dark photoperiod. This experiment was performed with three independent repetitions for each virus treatment and both evaluation time points. After 7 and 14 days, larvae were collected from each treatment and subjected to total DNA isolation ut supra. DNA samples were used for the identification of *egt* type I to V by partial PCR amplification.

### 2.10. Crowding Experiments with Phop-IT

Groups of different numbers (5, 10, 15, 20, 30 or 40) of neonate larvae of the *P. operculella* population Phop-IT were placed on a potato disc (ᴓ 43 mm and 5 mm thickness). Each of the potato slices was kept in a Petri dish and incubated at 28 °C with a 16/8 h light/dark photoperiod. The numbers of non-symptomatic larvae, symptomatic virus infected larvae and pupae were recorded after 13 days. For each tested number of neonate larvae, eight independent replicates were conducted.

### 2.11. Whole Genome Sequencing of Isolates

About 150 ng/sample of genomic DNA of PhopGV-R, -CR3.1 and -GR1.1 were sent for whole genome sequencing (StarSEQ GmbH, Mainz, Germany) with 150 bp read length and paired-end options [35]. Library preparation and bioinformatic analysis of sequencing data followed the protocol as described by Larem et al. [35].

## 3. Results

### 3.1. Identification of a Covert Infection in Phop-IT

**Figure 1:**
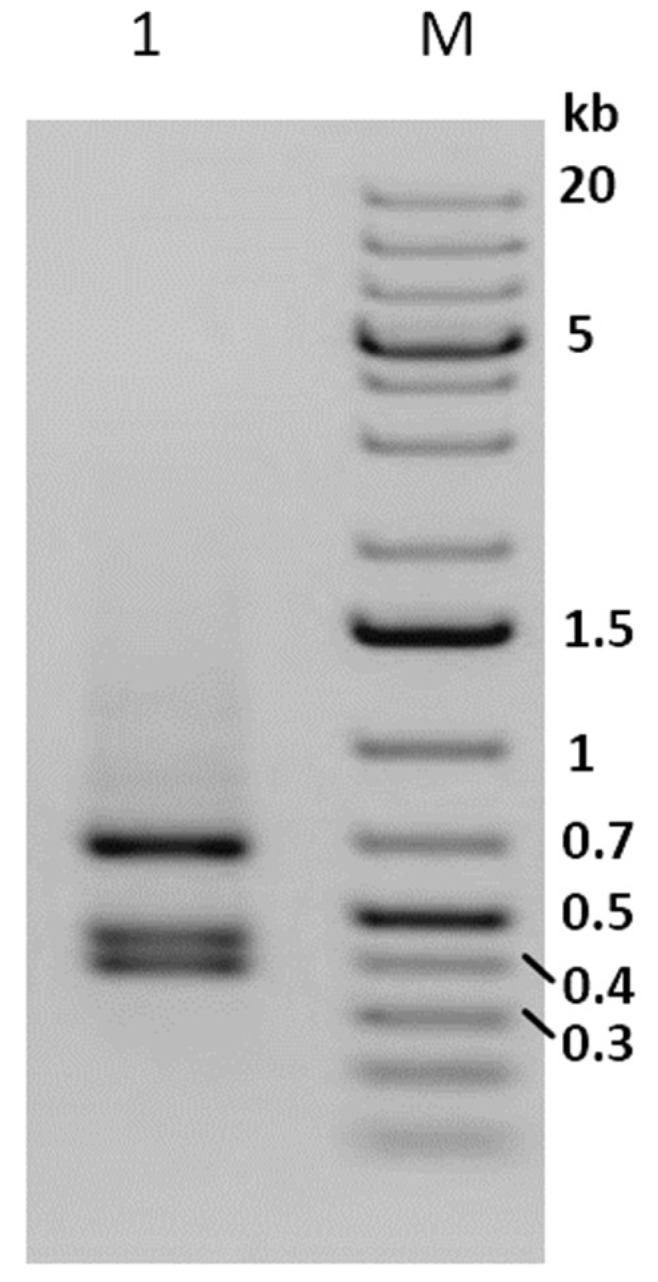
Agarose gel (1%) electrophoresis of the AluI digest of the *egt* PCR product from purified DNA of collected single larvae from the insect population Phop-IT (lane 1). Size marker (lane M) 1 kb plus ladder. A negative image of the stained gel is shown for better visualization of the PCR fragments.

After 48 weeks (12 generations) of maintaining Phop-IT in the laboratory, sporadic occurrence of virus infected larvae was observed, suggesting the presence of a covert virus in the rearing. During a time period of 20 weeks, covering four generations of Phop-IT rearing, 80 symptomatic L3/L4 larvae showing phenotypical signs of baculovirus infection, such as lethargic behaviour, whitish color and tumid appearance, were picked up out of several thousand healthy-looking larvae. Detection of PhopGV was done by DNA purification from single larvae followed by DNA purification and a PCR with specific primers for PhopGV *egt* gene fragment. The PCR results confirmed the phenotypical observation of a virus infection of the collected *P. operculella* larvae. The size of the amplified *egt* fragment was 1557 bp, which included a 204 bp flanking region to the *egt* ORF. Thus, the *egt* coding region was predicted to be 1353 bp, indicating an *egt* type II of the isolated PhopGV according to Carpio et al. [32]. This finding was corroborated by digesting the PCR product with AluI, resulting in three AluI fragments of 413 bp, 462 bp and 682 bp, as is typical for *egt* type II (Figure 1). This covert virus in the rearing of Phop-IT was termed PhopGV-R. When two additional *P. operculella* populations originating from a potato storage room in Tunisia and a field collection from Egypt were tested for the presence of PhopGV, the same pattern as that shown in Figure 1, lane 1, was detected [45]. This finding indicated that stable covert infections were wide-spread in *P. operculella*, and no virus-free population could be identified in our experiments. When DNA was isolated from healthy-looking larvae, eggs, pupae or adults tested by PCR for PhopGV, no virus could be detected with that method [45]; thus, it is not clear how the internal virus is carried.

### 3.2. Sequence Analysis of PhopGV-R

The 80 collected larvae were pooled and homogenized before further virus purification and DNA isolation. Purified DNA from the covert virus PhopGV-R was subjected to Illumina sequencing. The GC content of the sequenced isolate PhopGV-R was 35.7%, which was not different from the GC content of other PhopGV isolates. General information about the sequenced PhopGV-R is given in Table 1. With a genome length of 119,080 bp, encoding 130 ORFs (Supplementary Table S1), PhopGV-R was close to the genome length of 119,061 bp for PhopGV-GR1.1 and was one of the longer PhopGV sequences, ranging between 118,355 bp (PhopGV-CR3.1) and 119,177 bp (PhopGV-IT1.1), which was the current range of the genome lengths of sequenced PhopGV isolates [35]. Sequence analyses confirmed the *egt* (ORF 129) length of 1353 bp (type II) of PhopGV-R. The two other PhopGV isolates used in this study, PhopGV-GR1.1 and PhopGV-GR1.1, showed *egt* ORFs of 1053 bp (type III) and a mixture of both types, respectively [17,32,35].

### 3.3. Single Nucleotide Polymorphisms (SNPs)

For the detection of SNPs, only positions with a variable frequency above 5% were included in this analysis; SNPs with lower frequency were considered as minor variation and were not further considered. Compared to the published reference sequence PhopGV-1346, the analyzed sample PhopGV-R showed a total of 97 SNP positions (Supplementary Table S2). Eighty SNPs were located within open reading frames (ORF), of which 46 SNPs resulted in an amino acid change. Forty-two SNPs were located inside ORFs with known function, of which 21 SNPs resulted in amino acid changes in 14 different ORFs (Table 2). Nine out of these 14 ORFs (*p49*, *p74*, *lef-1*, *helicase-1*, *lef-4*, *vp39*, *vlf-1*, *DNApol* and *lef-8*) belonged to the core gene family, which includes ORFs shared by all baculoviruses [46].

Changes in the nucleotide sequence, leading to altered amino acid sequences of the resulting proteins with known function, were found in four genes involved in DNA replication (*lef-1*, *helicase-1*, *DNApol* and *lef-3*), in three genes involved in transcription (*pe38*, *lef-4* and *lef-8*), one gene for oral infectivity (*p74*), one for cell-to-cell infectivity (*efp*), two auxiliary genes (*MP-nase* and *sod*) and three genes involved in packing, assembly and release (*p49*, *vp39* and *vlf-1*) (Table 2) [47]. All these SNPs, resulting in amino acid changes, were also found in PhopGV-CR3.1, but only eleven of them occurred in the majority number group of the supporting reads, the remaining ten SNPs occurred in the minority number group of reads. PhopGV-GR1.1 shared six SNPs with PhopGV-R, which were all supported by the majority number of reads (Table 2).

### 3.4. Insertions and Deletions (Indels)

**Figure 2:**
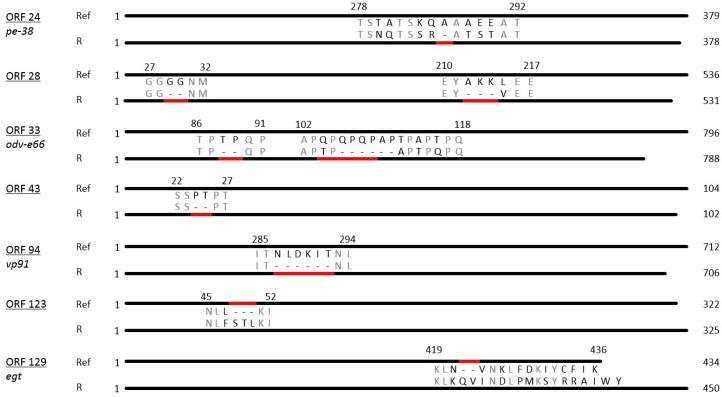
Diagrammatic representation of amino acid sequences representing ORFs with indels in PhopGV-R aligned with the respective ORFs from the reference isolate NC004062. The total amino acid length of each ORF is shown to the right, while the numbers above each insertion/deletion region represent the corresponding amino acids from the aligned reference sequence. Deletions/insertions are indicated by a red bar.

PhopGV-R showed indel mutations in relation to the reference isolate PhopGV-1346 (NC004062). These indels comprised seven deletions and two insertions allocated to seven different ORFs (24, 28, 33, 43, 94, 123 and 129) of the PhopGV genome (Figure 2). The distribution of these indels is typical for an isolate of genome group I of PhopGV [35], but two indels, namely ORF 93 and ORF 123, were additionally observed for PhopGV-R. Four ORFs coded for proteins of known function, namely ORF 24 (*pe-38*), a gene involved in transcription, ORF 33 (*odv-e66*), coding for a structural protein involved in oral infectivity, ORF 94 (*vp91*), involved in packing assembly and release, and ORF 129 (*egt*), which is an auxiliary gene [47,48]. ORF 24 (*pe-38*) showed a deletion of an alanine amino acid (aa) residue (ΔA), ORF 33 (*odv-e66*) had two deletions (ΔTP and ΔQPQPAP), ORF 94 (*vp91*) one deletion (ΔNLDKIT). ORF 129 (*egt*) contained one insertion (+QV), resulting in an *egt* type II with a length of 434 bp (Figure 2) [38]. Further indels were present in three ORFs, coding for proteins with unknown function: two deletions (ΔGG and ΔAKK) in ORF 28, one deletion in ORF 43 (ΔPT) and one insertion in ORF 123 (+STL). Five indels were also present in PhopGV-CR3.1 (ORF 24, 28, 33, 43 and 129). PhopGV-GR1.1 showed no concurrence with the indels of PhopGV-R displayed in Figure 2.

### 3.5. Sod Frequency

**Figure 3:**
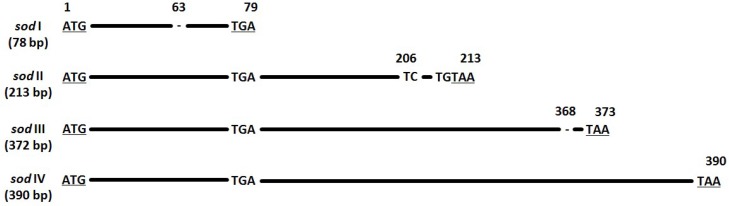
Nucleotide alignment of the four *sod* types (I–IV). Nucleotide positions and differences are displayed in relation to *sod* type IV (390 bp). Start and stop codons are underlined.

The protein superoxide dismutase (*sod*) generally has a function of removal of radicals which produce cell-damaging superoxide. It is present in almost all aerobic organisms [49], and also in almost all lepidopteran baculovirus genomes [4]. There is no evidence that baculovirus-coded *sod* has the same function as the *sod* in other species, and further, that a functioning baculovirus *sod* gets expressed [50]. Different PhopGV isolates have *sod* ORFs with varying lengths [35]. The *sod* (ORF 54) of PhopGV-R was detected in two variations: *sod* I (78 bp) and *sod* IV (390 bp), with a frequency of of 0.09 and 0.91, respectively (Figure 3). The other two *sod* types occurring in PhopGV, *sod* II (213 bp) and *sod* III (372 bp) [35], were not present for PhopGV-R. 

### 3.6. Virus Propagation of PhopGV-CR3 Resulted in Double Infections

**Figure 4:**
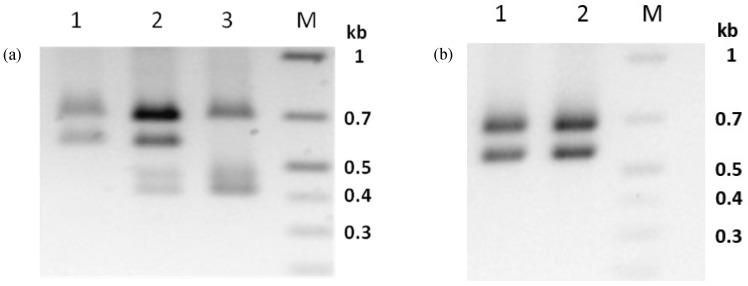
Agarose gel (1%) electrophoresis of the AluI digested PCR product *egt* of PhopGV-CR3 (**a**) DNA from PhopGV-CR3 occlusion bodies, *egt* type III (1), DNA extracted from CR3 infected Phop-IT single larva (CR3.1), mixture of *egt* type II and III (2) and DNA extracted from PhopGV-R infected single larva, *egt* type II (3). AluI digested PCR product *egt* of PhopGV-GR1 (**b**) DNA from PhopGV-GR1 occlusion bodies, *egt* type III (1) and DNA extracted from PhopGV-GR1 infected Phop-IT single larva (PhopGV-GR1.1), *egt* type III (2). M = GeneRuler 1 kb plus DNA ladder (Thermo Scientific). A negative image of the stained gel is shown for better visualization of the PCR fragments.

Propagation of isolate PhopGV-CR3 in neonate larvae of Phop-IT and purification of OB from 80 pooled, infected larvae resulted in the sample PhopGV-CR3.1. Whereas PhopGV-CR3 was considered a 100% pure *egt* type III, PCR analysis of the *egt* gene fragment of the propagated virus sample PhopGV-CR3.1 revealed a mixture of *egt* type III, as typical for PhopGV-CR3 and *egt* type II, pointing to the presence of PhopGV-R. Whole DNA extraction from a single Phop-IT larva infected with PhopGV-CR3 showed that a single larva could become co-infected by PhopGV-CR3 and PhopGV-R (Figure 4a). Indeed, complete genome sequencing of PhopGV-CR3.1, the purified virus DNA of 80 pooled larvae infected with PhopGV-CR3, confirmed a mixture of both viruses. The genotype quantification was based on the different number of AluI cleavage sites (AG’CT) on *egt* (ORF 129). PhopGV-CR3 has one recognition site for AluI, whereas PhopGV-R, in contrast, bears two recognition sites for the *egt* PCR product. The total number of reads was counted for the occurrence of this second AluI site to ensure a quantification of these two isolates in a mixture. The coverage of 1203 reads supported 962 times the second AluI site, which is present in PhopGV-R (*egt* type II) but not in PhopGV-CR3 (*egt* type III). It was estimated that inoculation of 80 Phop-IT neonate larvae with 100% PhopGV-CR3 resulted in a progeny of 20% PhopGV-CR3 and 80% PhopGV-R virus propagation (see below). The *egt-*based quantification of genotypes could be confirmed by *sod* type analysis; isolates which showed a mixture of different *egt* types also showed different *sod* types (ORF 54). Thus, a recombination of *egt* (ORF 129) only during propagation in Phop-IT is not plausible. A benefit of AluI site quantification was that it could be visualized also on a gel after digestion of *egt* PCR products (Figure 4).

For this study, *egt* type II with 1353 bp in length and type III with 1053 bp were of further interest, while other types (I, IV and V) did not occur. *Egt* type II showed three AluI fragments of 413 bp, 462 bp and 682 bp in length (Figure 4a, lane 3), whereas *egt* type III had two AluI fragments with 575 bp and 682 (Figure 4a, lane 1). A mixture of both types was generating four fragments (Figure 4a, lane 2). The sum of the AluI fragment sizes was larger than the ORF length of *egt*, because the *egt* PCR products covered up- and down-stream flanking sequences of 204 bp.

### 3.7. PhopGV-GR1 Suppresses Replication of PhopGV-R

Whereas infection of Phop-IT larvae with PhopGV-CR3, led to a mixed infection with a majority of PhopGV-R in the viral progeny, a different result was obtained when PhopGV-GR1 was propagated in Phop-IT. The resulting virus progeny PhopGV-GR1.1 was free of any contamination with PhopGV-R. PhopGV-GR1 showed the same *egt* type III before and after propagation in Phop-IT single larva, i.e., the *egt* PCR patterns of PhopGV-GR1 and -GR1.1 were identical (Figure 4b). Lack of replication of PhopGV-R during propagation of -GR1 is corroborated by the lack of PhopGV-R after infection of 80 Phop-IT larvae with PhopGV-GR1, resulting in PhopGV-GR1.1, to produce DNA for genome sequencing and the virus preparation of the pooled larvae did not show any contamination with covert PhopGV-R after an infection with PhopGV-GR1 (see below), suggesting that covert PhopGV-R is suppressed by -GR1. A simple recombination of *egt* (ORF 129) can be excluded because analysis of the whole genome sequencing data of PhopGV-GR1.1 showed only one *sod* type, a contamination with PhopGV-R would introduce a second *sod* type, and PhopGV-GR1.1 shows an insertion of 150 nt between ORF 109 and ORF 110, which can be used to differentiate from PhopGV-R [35].

**Figure 5:**
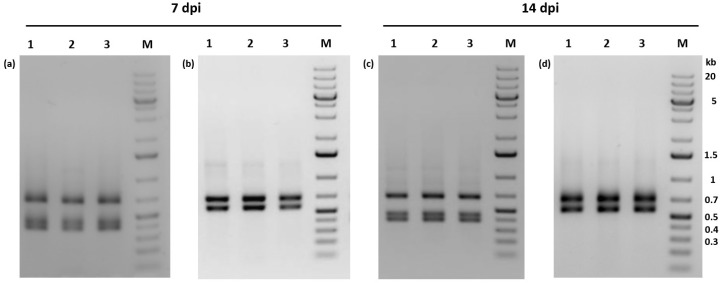
Agarose gel electrophoresis (1%) of AluI digests of egt-specific PCR fragments obtained from infections of Phop-IT larvae using single virus and co-infections. Virus progeny were analyzed 7 and 14 dpi. Larvae were per orally infected with either PhopGV-R (**a**,**c**) or a 1:1 mixture of PhopGV-R and PhopGV-GR1.1 (**b**,**d**). Detection experiments were performed in triplicate (lanes 1–3), with each replicate consisting of 20 infected larvae. M = GeneRuler 1 kb plus DNA ladder (Thermo Scientific). Negative images of the stained gels were shown for better visualization of the PCR fragments.

To study whether PhopGV-R can also be suppressed by PhopGV-GR1 (or –GR1.1) during peroral infection, 60 neonate larvae of Phop-IT were infected with either the purified PhopGV-R, a mix (1:1) of PhopGV-R and PhopGV-GR1.1, or water. This experiment was performed twice, and larvae were collected after 7 dpi and 14 dpi. A whole DNA extraction was performed, followed by standard PCR with *egt*-specific primers. The PCR fragments were then digested with AluI to confirm the identity of the isolated virus (Figure 5). Larvae infected with PhopGV-R showed only *egt* type II restriction patterns, confirming peroral infectivity of PhopGV-R (Figure 5a,c). Single larvae infected with PhopGV-GR1.1 gained the same restriction pattern as observed in Figure 4b after 7 and 14 dpi [51]. However, larvae infected with a mixture of PhopGV-R and PhopGV-GR1.1 showed only restriction patterns specific for *egt* type III. Neither *egt* type II alone nor mixtures of type II and III were observed. The 60 mock control single larva did not show any virus infection [51].

### 3.8. Co-Propagation of PhopGV-CR3 and PhopGV-GR1

**Figure 6:**
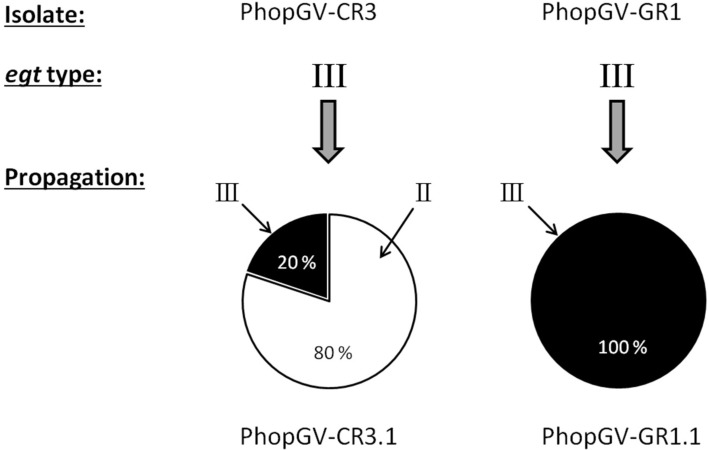
Quantification (%) of the *egt* types of PhopGV-CR3 and -GR1, based on the de novo assembled reads of the Illumina sequencing data after propagation in *P. operculella* population Phop-IT. The *egt* type is indicated with roman numbers.

Upon propagation in Phop-IT larvae, *egt* type III of isolate PhopGV-CR3 was replaced by *egt* type II. This result was due to replication of the covert virus PhopGV-R, which is an *egt* type II virus (Figure 6). After propagation, the resulting virus progeny PhopGV-CR3.1 consisted of an additional *egt* type (II), whereas PhopGV-GR1.1 kept the same *egt* type.

### 3.9. Crowding of Larvae Had No Effect on Overt Virus Infections

To test whether crowding of larvae of Phop-IT had an effect on the development of overt infections, 5–40 neonate larvae were placed on a potato disc and the number of symptomatic larvae, non-symptomatic larvae and pupae was recorded after 13 days. Symptomatic in this particular context means that signs of overt virus infection were present. The rate of symptomatic lethally infected larvae ranged between 1.1% and 4.1% with crowding, but the differences were not significant (Table 3). Only the pupation rate was significantly reduced at higher densities of 40 individuals per potato disc, compared to low densities of 5 to 15 larvae per disc (*t*-test, Bonferroni, *p* = 0.037). In addition, the total number of survivors was significantly reduced by 23% (*t*-test, Bonferroni, *p* = 0.029), when crowdings of 40 and 5 larvae per disc were compared. No significant differences were observed for the number of non-symptomatic larvae and the number of symptomatic virus infected larvae.

## 4. Discussion

Persistent covert infections are supposed to be a viral strategy for transmission and the answer to fluctuating host populations [13,28]. Persistent covert baculovirus infections have been observed not only for lepidopteran laboratory colonies but also in field populations [13,27]. In this study, a stable covert infection inside the laboratory-reared colony Phop-IT was detected. A covert infection with PhopGV was also detected in two other *P. operculella* populations from Tunisia and Egypt [45]. Hence, covert infections of *P. operculella* colonies seem to be common. It has been hypothesized that persistent infections cannot be cured, and are likely the result of surviving virus challenge [1,22,27]. It is possible that covert virus infections are the rule and not the exception for insect populations, and occasionally such a covert infection may become overt. A crowding effect was described in the literature for being a stressor for covert infected insect colonies to start an active overt infection in *Trichoplusia ni* populations [14], *Colias philodice eurytheme* and *Junonia coenia* [29], *Mamestra brassicae* [20,52] and *Thaumatotibia leucotreta* [18].

There was no significant connection between crowding of insects and a triggered overt infection for PhopGV-IT, though there was a weak increase of virus outbreak with increasing number of larvae per potato disc. During the crowding experiment, virus-caused mortality stayed below 5%, but there was a significant reduction in the total number of living individuals if a low- or high-density number of individuals were placed on each potato disc. This could not be clearly assigned to virus activity, because at high larval density, food competition could also have had an effect on larval development. The extent of pupation was significantly reduced at higher densities compared to low densities of Phop-IT larvae per disc. This effect could be driven by either virus influence, food competition or stress at higher population densities.

The activation of covert infections to overt infections by infection with a second virus are reported for different baculoviruses; the activating virus can be either homologous or heterologous, belonging to another baculovirus species [11,13,15,20,28,53]. Such a triggering effect by a homologous virus isolate was also observed for PhopGV in this study when individuals of Phop-IT were infected by PhopGV-CR3 and the covert virus PhopGV-R was replicated at a high level. This effect was first noticed by the presence of the *egt* type II when PhopGV-CR3 was propagated; the original *egt* type III of PhopGV-CR3 was present in only a minority of cases for the propagated PhopGV-CR3.1. 

Surprisingly, PhopGV-GR1 did not activate the covert virus PhopGV-R during propagation in Phop-IT larvae. The resulting PhopGV-GR1.1 was free of contamination with PhopGV-R. Even when larvae were inoculated with a 1:1 mixture of PhopGV-R and PhopGV-GR1.1, only PhopGV-GR1.1 was detected in the progeny. This finding suggested that not every homologous infection activates the covert virus in the Phop-IT population. In contrast, there must be a mechanism blocking the replication of PhopGV-R when larvae become infected with PhopGV-GR1, but too little is known at this time. 

Analyses of ORF 54 (*sod*) showed that PhopGV-R is composed of at least two different genotypes. This observation raises the question as to whether both genotypes play a role in the covert infection of Phop-IT. In contrast, the overt infecting PhopGV-GR1.1 has only one *sod* type. Whether *sod* can be used as a selection marker for virulence has not yet been demonstrated and needs further investigation with respect to its real function during the infection process.

All SNPs specific for PhopGV-R occurred either in a majority or minority of reads in PhopGV-CR3.1 (Table 2). This can be explained by the composition of PhopGV-CR3.1, consisting of 80% PhopGV-R. The finding that PhopGV-CR3.1 showed eleven majority SNPs and ten minority ones can be explained by the fact that PhopGV-R is heterogenic and PhopGV-CR3.1 consists of at least three different genotypes in mixture. 

For alphabaculoviruses, super-infection exclusion is connected with actin reorganization [54]. It has been unclear, until now, which mechanism is responsible for co-infection exclusion in connection with PhopGV isolates. Vertically transmitted pathogens may prevent co-infection [55], but the primarily vertical transmitted PhopGV-R did not show a co-infection exclusion. Rather, PhopGV-R was excluded by the horizontally transmitted PhopGV-GR1. In contrast to super-infection exclusion, virus co-infections can occur in single larva [56,57,58]. A co-infection of single Phop-IT larvae was observed for PhopGV-R and PhopGV-CR3. It appears that PhopGV isolates interact in different ways if occurring in the same host individual; some PhopGV isolates exclude each other, whereas others tolerate each other.

It might be a long-term survival strategy of some less virulent pathogens to adapt or manipulate the life cycle of the host [59]. In contrast, highly virulent parasites and pathogens are at risk of becoming extinct (“fade-out”), according to ecological models, because of a rapid exhaustion of the pool of susceptible hosts [60,61,62]. For insects with low densities, overt baculovirus infections are rare [24], and horizontal virus transmission may be inefficient because of the low likelihood that a non-infected larva encounters an infected one [26]. If the virus becomes exposed to UV radiation outside the host, the infectivity of the virus can be reduced 10- to 100-fold within a few days [63,64]. Considering UV inactivation of OB and low host densities, selection for low virulence may increase the survival chance of the virus population, leading to persistent vertically transmitted covert baculovirus infections.

Insect communities, including *P. operculella,* may carry covert baculovirus infections which influence the host population dynamically by interaction of endogenous and exogenous viruses, either by tolerating, mixing or blocking each other when larvae are perorally infected. This finding needs to be considered to exploit the full potential of baculovirus application for plant protection reasons. It can be speculated that such covert infections of insect populations may contribute to the success and failure of baculovirus applications in the field, depending of the nature of interaction between the covertly present virus and the applied virus genotype, as observed in this study for the two isolates PhopGV-CR3 and PhopGV-GR1.

## Figures and Tables

**Table 1:** Characteristics of consensus sequence (>50% majority) of the sequenced genomes of the PhopGV-R isolate and two additional PhopGV isolates used for this study.

Isolate (PhopGV-)	Origin	Reads Assembled	Coverage/nt	Consensus Seq. Length	Accession No.	Source/Reference
R	Darmstadt	1,163,983	1284	119,080	MK033575	JKI
CR3.1	Costa Rica	936,612	1187	118,355	MK033565	UPNA/[38]
GR1.1	Greece	1,647,866	2076	119,061	MK033567	Hellafarm

**Table 2:** Single nucleotide polymorphisms (SNPs) in PhopGV-R open reading frames (ORF) with known functions in relation to the reference isolate PhopGV-1346 (NC004062). SNPs occurred in the majority of reads (>51%) with one exception (*) where a minority of 22.4% was detected. The occurrence of the respective SNPs in PhopGV-CR3.1 and PhopGV-GR1.1 is indicated with (+) for majority and (−) for minority number of reads supporting this SNP.

ORF	Encoding Gene	Direction	Position	Change (nt)	Change (aa)	Present	
						CR3.1	GR1.1
13	*p49*	reverse	9514	A -> G	Tyr -> His	+	
24	*pe38*	reverse	16,657	T -> G	Lys -> Ans	−	
27	*efp*	forward	21,377	C -> A	Thr -> Lys	−	
41	*MP-nase*	reverse	35,905	C -> T	Asp -> Asn	+	
54	*sod*	reverse	45,394	G -> A	Leu -> Phe	−	
54	*sod*	reverse	45,586	A -> T	Cys -> Ser	+	+
55	*p74*	reverse	47,389	C -> A	Arg -> Ile	−	
66	*lef-1*	reverse	56,063	C -> T	Cys -> Tyr	−	
66	*lef-1*	reverse	56,420	A -> G	Val -> Ala	−	
82	*helicase-1*	forward	68,953	C -> G	Thr -> Arg	+	
87	*lef-4*	reverse	75,799	G -> A	Ala -> Val	−	
87	*lef-4*	reverse	76,009	C -> A	Ser -> Ile	+	+
87	*lef-4*	reverse	76,284	C -> T	Met -> Ile	+	+
87	*lef-4*	reverse	76,364	T -> C	Tyr -> Ala	−	
88	*vp39*	forward	77,124	T -> G	Ser -> Ala	+	
99	*vlf-1*	forward	85,078	T -> G	Tyr -> Asp	+	+
99	*vlf-1*	forward	85,232	A -> C	Asn -> Thr	−	
103	*DNApol*	reverse	87,530	G -> C	Gln -> Glu	+	+
105	*lef-3*	reverse	93422	G -> C	Asn -> Lys	+	+
105	lef-3(*)	reverse	94,066	A -> T	Leu -> Met	−	
121	*lef-8*	reverse	111,135	C -> T	Gly -> Arg	+	

**Table 3:** Effect of crowding of Phop-IT larvae on a potato disc (ᴓ 43 mm) at 28 °C after 13 days. Larvae were observed to show symptomatic virus signs (s) or were not symptomatic. The letters (abc) after a number indicate statistically significant differences (*t*-test, Bonferroni) within the different categories. *N* = number of independent replicates, *n* = total number of tested larvae.

Larvae/Disc	5	10	15	20	30	40
**N**	8	8	8	8	8	8
**n**	100	180	285	380	540	680
**% Larvae (s)**	1.1 ± 4.6	1.7 ± 3.9	1.8 ± 3.8	2.1 ± 3.8	3.2 ± 4.4	4.1 ± 4.2
**% Larvae (not s).**	9 ± 16.5	4.4 ± 12.5	4.6 ± 5.9	2.6 ± 4.5	2.8 ± 3.2	7.2 ± 9.8
**% Pupae**	67 ± 29.2a	70.6 ± 21a	68.1 ± 15.3a	59 ± 15.8ab	55.7 ± 10.3ab	50.5 ± 11.5bc
**% Living**	77 ± 25.4a	76.1 ± 17.9ab	73.7 ± 15.8ab	63.7 ± 16.2ab	61.8 ± 9.9ab	59 ± 11.5b

## Supplementary Materials

The following are available online at https://www.mdpi.com/1999-4915/11/4/337/s1, Table S1: Annotation of PhopGV-R compared to the reference isolate PhopGV-1346. Table S2: List of the SNP positions of PhopGV-R compared to the reference isolate PhopGV-1346.
