# Supplementary material for: Effects of a Covert Infection with Phthorimaea operculella granulovirus in Insect Populations of Phthorimaea operculella
Source: Viruses. 2019 Apr 9;11(4):337. doi: 10.3390/v11040337 (PMC6520744; doi:10.3390/v11040337)
Supplement: Supplementary file 1 [file viruses-11-00337-s001.pdf]

Supplementary Materials:

Table S1: Annotation of PhopGV-R compared to the reference isolate PhopGV-1346. Differences in the amino acid length are highlighted with bold red numbers. The dedicated reference was highlighted with bold blue numbers.

		**PhopGV-1346**			**PhopGV-R**		
ORF	Name	Position	length (bp)	length (aa)	Position	length (bp)	length (aa)
1	*granulin*	1 > 747	747	248	1 > 747	747	248
2	*P78/83*	883 < 1404	522	173	883 < 1404	522	173
3	*pk-1*	1385 > 2221	837	278	1385 > 2221	837	278
4		2357 < 2929	573	190	2386 < 2958	573	190
5		2922 > 3161	240	79	2951 > 3190	240	79
6	*ie-1*	3538 < 4818	1281	426	3567 < 4847	1281	426
7		4842 > 5459	618	205	4871 > 5488	618	205
8		5479 < 5850	372	123	5508 < 5879	372	123
9	*bro*	6003 > 7079	1077	358	6064 > 7140	1077	358
10		7181 > 7351	171	56	7242 > 7412	171	56
11		7638 > 7877	240	79	7699 > 7938	240	79
12	*ODV-e18*	8027 < 8278	252	83	8088 < 8339	252	83
13	*P49*	8279 < 9559	1281	426	8340 < 9620	1281	426
14		9769 < 10386	618	205	9831 < 10448	618	205
15		10475 < 11434	960	319	10529 < 11488	960	319
16	*ODV-e56*	11464 < 12519	1056	351	11518 < 12573	1056	351
17		12634 > 12822	189	62	12688 > 12876	189	62
18		13124 < 13285	162	53	13178 < 13339	162	53
19		13354 < 13827	474	157	13408 < 13881	474	157
20		13890 > 14798	909	302	13944 > 14852	909	302
21		14824 > 15264	441	146	14878 > 15318	441	146
22		15315 > 15680	366	121	15369 > 15734	366	121
23		15754 > 16068	315	104	15808 > 16122	315	104
24	*PE-38*	16369 < 17508	1140	**379**	16423 < 17559	1137	**378**
25		18497 > 19639	1143	380	18546 > 19688	1143	380
26		20524 > 21063	540	179	20573 > 21112	540	179
27	*efp*	21193 > 22980	1788	595	21242 > 23029	1788	595
28		23479 > 25089	1611	**536**	23528 > 25123	1596	**531**
29		25295 < 25957	663	220	25293 < 25955	663	220
30		26339 < 26902	564	187	26336 < 26899	564	187
31		26906 > 27439	534	177	26903 > 27436	534	177
32		27436 < 29463	2028	675	27433 < 29460	2028	675
33	*ODV-e66*	29470 < 31860	2391	**796**	29467 < 31833	2367	**788**
34		31911 > 32225	315	104	31884 > 32198	315	104
35		32360 < 32653	294	97	32333 < 32626	294	97
36		32826 > 33161	336	111	32826 > 33161	336	111
37	*lef-2*	33154 > 33696	543	180	33154 > 33696	543	180
38		33951 > 34208	258	85	33951 > 34208	258	85
39		34229 < 34573	345	114	34229 < 34573	345	114
40		34720 < 35073	354	117	34720 < 35073	354	117

41	*mp-nase*	35132 < 36541	1410	469	35132 < 36541	1410	469
42	*p13*	36546 > 37379	834	277	36546 > 37379	834	277
43		37402 > 37716	315	**104**	37402 > 37710	309	**102**
44		37732 > 38832	1101	366	37726 > 38826	1101	366
45		39075 < 39278	204	67	39070 < 39273	204	67
46		39303 > 41735	2433	810	39298 > 41730	2433	810
47		41668 < 42576	909	302	41663 < 42571	909	302
48		42588 > 42731	144	47	42583 > 42726	144	47
49	*v-ubi*	42825 < 43109	285	94	42820 < 43104	285	94
50		43229 > 44248	1020	339	43224 > 44243	1020	339
51		44260 > 44406	147	48	44255 > 44401	147	48
52	*PP31*	44421 < 45116	696	231	44416 < 45111	696	231
53	*lef-11*	45100 < 45366	267	88	45095 < 45361	267	88
54	*sod*	45531 < 45850	390	129	45446 < 45835	390	129
55	*p74*	46284 < 48260	1977	658	46270 < 48246	1977	658
56		48436 < 48852	417	138	48422 < 48838	417	138
57		49042 > 49458	417	138	49028 > 49444	417	138
58		49702 > 50475	774	257	49688 > 50461	774	257
59		50673 < 51266	594	197	50659 < 51252	594	197
60		51354 < 51872	519	172	51340 < 51858	519	172
61	*p47*	51966 > 53165	1200	399	51952 > 53151	1200	399
62		53247 > 53894	648	215	53233 > 53880	648	215
63	*p24*	54103 > 54606	504	167	54089 > 54592	504	167
64		54608 > 55165	558	185	54594 > 55151	558	185
65	*p38.7*	55443 < 56000	558	185	55430 < 55987	558	185
66	*lef-11*	55894 < 56595	702	233	55881 < 56582	702	233
67		56788 > 58356	1569	522	56775 > 58343	1569	522
68		58379 > 58543	165	54	58366 > 58530	165	54
69	*fgf-1*	58591 < 59310	720	239	58578 < 59297	720	239
70		59452 < 59769	318	105	59439 < 59756	318	105
71		60034 > 60492	459	152	60021 > 60479	459	152
72	*lef-6*	60579 < 60863	285	94	60566 < 60850	285	94
73	*dbp*	61039 < 61872	834	277	61026 < 61859	834	277
74		62051 < 62578	528	175	62038 < 62565	528	175
75		62730 > 63869	1140	379	62717 > 63856	1140	379
76		64031 > 64357	327	108	64032 > 64358	327	108
77	*BV/ODV-c42*	64439 > 65578	1140	379	64440 > 65579	1140	379
78	*p6.9*	65595 > 65759	165	54	65596 > 65760	165	54
79	*lef-5*	65908 < 66630	723	240	65910 < 66632	723	240
80		66583 > 67482	900	299	66585 > 67484	900	299
81		67991 < 68479	489	162	67993 < 68481	489	162
82	*helicase 1*	68463 > 71864	3402	1133	68465 > 71866	3402	1133
83	*ODV-e25*	72037 < 72681	645	214	72039 < 72683	645	214
84		72812 < 73297	486	161	72814 < 73299	486	161
85		73324 > 74082	759	252	73326 > 74084	759	252
86	*iap-Op1*	74312 < 75049	738	245	74313 < 75050	738	245
87	*lef-4*	75075 < 76400	1326	441	75076 < 76401	1326	441

88	*vp39*	76455 > 77336	882	293	76456 > 77337	882	293
89	*ODV-ec27*	77442 > 78305	864	287	77443 > 78306	864	287
90		78469 > 78621	153	50	78470 > 78622	153	50
91		78629 > 78784	156	51	78630 > 78785	156	51
92		78733 < 79944	1212	403	78734 < 79945	1212	403
93		79931 > 80224	294	97	79932 > 80225	294	97
94	*vp91*	80297 < 82435	2139	**712**	80298 < 82418	2121	**706**
95	*tlp20*	82410 > 82763	354	117	82393 > 82746	354	117
96		82747 > 83418	672	223	82730 > 83401	672	223
97	*gp41*	83300 > 84157	858	285	83283 > 84140	858	285
98		84423 > 84680	258	85	84406 > 84663	258	85
99	*vlf-1*	84637 > 85782	1146	381	84620 > 85765	1146	381
100		85976 > 86233	258	85	85959 > 86216	258	85
101		86281 > 86718	438	145	86264 > 86701	438	145
102		86912 < 87277	366	121	86895 < 87260	366	121
103	*DNApol*	87378 < 90443	3066	1021	87361 < 90426	3066	1021
104	*desmoplakin*	90448 > 92703	2256	751	90431 > 92686	2256	751
105	*lef-3*	93266 < 94315	1050	349	93249 < 94298	1050	349
106		94281 > 94658	378	125	94264 > 94641	378	125
107		94727 > 95266	540	179	94710 > 95249	540	179
108	*iap-Cp5*	95540 > 96355	816	271	95523 > 96338	816	271
109	*lef-9*	96371 > 97864	1494	497	96354 > 97847	1494	497
110	*fp*	98117 > 98572	456	151	97922 > 98377	456	151
111		98517 > 99008	492	163	98322 > 98813	492	163
112	*DNA ligase*	99106 < 100788	1683	560	98911 < 100593	1683	560
113	*helicase 2*	100892 < 102211	1320	439	100697 < 102016	1320	439
114	*Alk-exo*	102165 < 103364	1200	399	101970 < 103169	1200	399
115		103648 < 103962	315	104	103453 < 103767	315	104
116	*fgf-2*	104102 > 105265	1164	387	103907 > 105070	1164	387
117		105311 < 105511	201	66	105116 < 105316	201	66
118		105587 < 105745	159	52	105392 < 105550	159	52
119	*rr1*	106154 < 107983	1830	609	105959 < 107788	1830	609
120	*rr2a*	108134 > 109171	1038	345	107939 > 108976	1038	345
121	*lef-8*	109360 < 111843	2484	827	109128 < 111611	2484	827
122		112234 > 112635	402	133	112002 > 112403	402	133
123		112773 < 113741	969	**322**	112541 < 113518	978	**325**
124		113813 < 114010	198	65	113590 < 113787	198	65
125	*lef-10*	114014 > 114235	222	73	113791 > 114012	222	73
126	*vp1054*	114099 > 115085	987	328	113876 > 114862	987	328
127		115240 > 115413	174	57	115017 > 115190	174	57
128		115463 > 116488	1026	341	115240 > 116265	1026	341
129	*egt*	116818 < 118122	1305	**434**	116633 < 117985	1353	**450**
130	*me53*	118275 > 119168	894	297	118138 > 119031	894	297

Table S2. List of the SNP positions of PhopGV-R compared to the reference isolate PhopGV-1346. Positions with also the reference sequence supported are highlighted with a star (*).

Number	Positon	Sequence	Nucleotide Change
1	3,135	G	G -> A*
2	3,159	A	A -> T
3	5,475	C	C -> A
4	7,882	T	T -> A*
5	9,514	A	A -> G
6	10,250	C	C -> T
7	10,313	A	A -> G
8	11,262	T	T -> C
9	11,291	T	T -> C
10	11,869	A	A -> G
11	12,811	A	A -> G
12	13,287	T	T -> C
13	13,634	C	C -> T
14	15,219	G	G -> A
15	16,144	T	T -> A
16	16,657	T	T -> G
17	17,725	G	G -> T*
18	17,831	G	G -> A
19	17,957	T	T -> C*
20	18,456	T	T -> A*
21	18,547	G	G -> A
22	19,364	A	A -> G*
23	20,306	C	C -> T*
24	21,377	C	C -> A
25	22,707	T	T -> C
26	23,092	T	T -> C
27	23,766	A	A -> G
28	26,152	G	G -> T
29	26,689	C	C -> G
30	26,880	C	C -> T
31	27,907	C	C -> T
32	28,240	C	C -> T
33	28,469	A	A -> T
34	28,923	T	T -> C
35	29,046	T	T -> C*
36	29,237	G	G -> A
37	32,127	T	T -> C
38	34,630	T	T -> G
39	35,372	G	G -> T*
40	35,905	C	C -> T
41	38,070	T	T -> C
42	41,421	A	A -> T
43	44,853	C	C -> T
44	44,991	C	C -> T

45	45,015	T	T -> C
46	45,394	G	G -> A
47	45,490	C	C -> G*
48	45,581	C	C -> A
49	45,586	A	A -> T
50	45,704	A	A -> G
51	45,827	C	C -> T
52	45,895	C	C -> A
53	46,544	A	A -> G
54	47,389	C	C -> A
55	49,221	G	G -> A
56	50,800	C	C -> A
57	51,733	A	A -> T
58	54,691	T	T -> C
59	56,063	C	C -> T*
60	56,420	A	A -> G*
61	57,537	G	G -> A
62	58,287	C	C -> T
63	65,773	A	A -> C
64	67,084	C	C -> T
65	68,953	C	C -> G
66	69,428	T	T -> C
67	71,916	T	T -> A
68	71,924	A	A -> T
69	75,799	G	G -> A
70	76,009	C	C -> A
71	76,284	C	C -> T
72	76,364	T	T -> C*
73	77,124	T	T -> G
74	78,035	C	C -> T*
75	78,462	G	G -> A*
76	78,683	C	C -> A*
77	79,369	T	T -> C
78	81,581	A	A -> T*
79	85,078	T	T -> G
80	85,232	A	A -> C
81	86,223	T	T -> C
82	86,986	C	C -> T*
83	87,530	G	G -> C
84	89,064	G	G -> A
85	90,684	G	G -> A*
86	93,422	G	G -> C
87	94,066	A	A -> T*
88	94,879	T	T -> C
89	109,726	G	G -> A
90	110,512	C	C -> T
91	110,773	T	T -> A

92	110,863	T	T -> G
93	111,135	C	C -> T
94	112,780	T	T -> C
95	115,269	C	C -> T
96	115,605	A	A -> T
97	116,855	T	T -> C*
